# 

**Published:** 1888-05-19

**Authors:** 


### CONTENTS.

**Our Hospital System** . . . . . . . 101

**In and Out Among the Hospitals.** By a Secretary of Ten Years' Standing . . . . 102

**The Needs and Backbone of the Hospital System.** By the Right Hon. Lord Randolph Churchill . . . . 103

**Labourers' Wages and Labourers' Homes.** By the Right Hon. Earl Grey, K.G., G.C.M.G. . . . . 105

**Sailors and Anti-scorbutics** . . . . . 107

**The Editor's Letter-box** . . . . . 107

**Notes and News** . . . . . . . 108

**Everybody's Page** . . . . . . . 110

**Annotations.**—The Site of St. Mary's Hospital.—Cheap Houses for Ladies.—An Unsavoury Subject . . . . 111

**Patients' Contributions to Hospitals.** By Mr. W. Burdett-Coutts, M.P. . . . . 112

**An Ishmaelite.** By Leslie Keith, Author of "The Chilcotes," "East and West," etc. . . . . 113

**Student's Column** . . . . . . 115

**Amusements with Profit for all Ages.**—For Men and Women.—Children's Corner.—Puzzles, etc. . . . 116

**EXTRA SUPPLEMENT.—THE NURSING MIRROR.**

En Passant.—A Church Parade.—The Last Stage of Illness.—The Evils of Opiates.—"Nursing Notes."—Beef Tea.—East London Nursing Society.—A Nursing Heroine.—Every Woman a Nurse . . . . . xxi
The National Pension Fund for Nurses . . . . . xxii
Workhouse Nursing . . . . . . . xxii
Lectures . . . . . . . . xxiii
Notes and Queries.—Everybody's Opinion.—Appointments.—Examination Questions.—Vacancies . . . . xxiv
Exchange and Mart . . . . . . . xxiv

